# Left ventricular posterior wall hypertrophy leads to poor prognosis of hypertrophic obstructive cardiomyopathy in children: a cohort study

**DOI:** 10.1097/JS9.0000000000001862

**Published:** 2024-06-21

**Authors:** Shun Liu, Xiumeng Hua, Yiqi Zhao, Han Mo, Xiao Chen, Weiteng Wang, Yijing Li, Qian Zhao, Jun Yan, Jiangping Song

**Affiliations:** a Department of Cardiovascular Surgery, Fuwai Hospital, National Center for Cardiovascular Diseases, Chinese Academy of Medical Sciences and Peking Union Medical College; b State Key Laboratory of Cardiovascular Disease, Fuwai Hospital, National Center for Cardiovascular Diseases, Chinese Academy of Medical Sciences and Peking Union Medical College; c Beijing Key Laboratory of Preclinical Research and Evaluation for Cardiovascular Implant Materials, Animal Experimental Centre, Fuwai Hospital, National Centre for Cardiovascular Disease, Chinese Academy of Medical Sciences and Peking Union Medical College; d Department of Pediatric Cardiac Surgery, Fuwai Hospital, National Center for Cardiovascular Diseases, Chinese Academy of Medical Sciences and Peking Union Medical College, Beijing; e Shenzhen Key Laboratory of Cardiovascular Disease, Fuwai Hospital Chinese Academy of Medical Sciences, Shenzhen, People’s Republic of China

**Keywords:** cardiac computed tomography, children, genetic mutation, hypertrophic cardiomyopathy, prognosis, three-dimensional model

## Abstract

**Objective:**

The modified Morrow operation for hypertrophic obstructive cardiomyopathy (HOCM) in children has a favorable outcome, but some children still have a poor prognosis after the procedure. In this study, the authors aimed to investigate the application of cardiac computed tomography (CCT) to construct a three-dimensional (3D) model of the left ventricle (LV) and analyze the association between hypertrophy in different parts of the LV and poor prognosis.

**Methods:**

The authors retrospectively analyzed 57 children with HOCM from April 2015 to October 2022, among whom 16 underwent preoperative CCT examination. All children underwent the modified Morrow surgery in our center. The authors defined heart failure, malignant ventricular arrhythmia, and recurrent left ventricular outflow tract obstruction (LVOTO) as adverse events. The authors performed a retrospective Cox analysis and conducted genetic testing. A 3D model of the LV was built through the standard 17-segment method and analyzing the high-risk factors.

**Results:**

Seventeen (29.8%) had adverse events during follow-up. Multivariate Cox analysis revealed that genetic mutation (HR: 5.634, 95% CI: 1.663–19.086, *P*=0.005), Noonan syndrome (HR: 3.770, 95% CI: 1.245–11.419, *P*=0.019), preoperational systolic anterior motion (HR: 4.596, 95% CI: 1.532–13.792, *P*=0.007)and mid-ventricular obstruction (HR: 4.763, 95% CI: 1.538–14.754, *P*=0.007) were high-risk factors, suggesting that the degree of hypertrophy in the LV is associated with poor prognosis. By analyzing the CCT with a 3D model, children with poor prognosis have more hypertrophy in basal-inferior (*P*=0.014), mid-inferoseptal (*P*=0.044), and mid-inferior (*P*=0.017). It suggests that a more hypertrophied posterior left ventricular wall portends a worse prognosis.

**Conclusion:**

Even after modified Morrow surgery, the prognostic impact of genetic mutation remains significant. Moreover, the degree of hypertrophy of the posterior wall in the LV was also related to the postoperative prognosis through CCT combined with 3D technology. It provides surgeons guiding to evaluate the overall prognosis and the treatment plan before surgery.

## Graphical abstract

**Figure:**
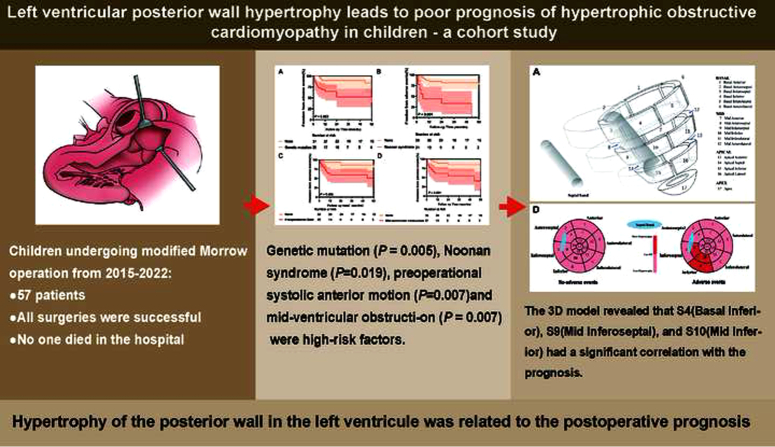

## Introduction

Highlights

- Systolic anterior motion and mid-ventricular obstruction were high-risk factors for poor prognosis.
- Cardiac computed tomography can well evaluate the degree of left ventricular hypertrophy in children with hypertrophic obstructive cardiomyopathy.
- Left ventricular posterior wall hypertrophy is a high-risk factor for poor prognosis in children with hypertrophic obstructive cardiomyopathy.

Hypertrophic obstructive cardiomyopathy (HOCM) is a cardiomyopathy characterized anatomically by asymmetric hypertrophy of ventricles. Asymmetric hypertrophy of the septum leads to left ventricular outflow tract obstruction (LVOTO) and systolic anterior motion (SAM)^1^. LVOTO occurs in approximately three-quarters of patients with hypertrophic cardiomyopathy (HCM) and is independently associated with an increased risk of HCM-related death and progression to heart failure (HF) or malignant arrhythmias^2,3^. Even after treatment with the standard modified Morrow operation, some structural abnormalities, such as biventricular abnormalities, have been reportedly found to correlate with poor prognosis^4^. Most children with HCM have a genetic mutation, including Friedreich’s ataxia, Noonan’s syndrome, and inborn errors of metabolism^5–7^. There are differences in ventricular hypertrophy and structure with different mutations, which lead to different prognoses^8,9^. However, we are still unclear about the prognostic implications of myocardial hypertrophy in specific locations of the left ventricle (LV).

Over time, rapid advances in computed tomography (CT) have led to cardiac computed tomography (CCT). It is an adjunctive examination capable of providing a comprehensive assessment of the anatomy and function of the intracardiac chambers of the heart. Although CCT is not the first test for the evaluation of cardiomyopathy disease, it provides a complete picture of the anatomy and function of the cardiac chambers. Its combination with three-dimensional (3D) technology allows for multiplanar reconstruction in the desired orientation^10,11^. Recent studies confirmed that CCT is an attractive imaging tool for characterizing LV myocardial disease based on gross morphology, function, and enhancement as well as extracellular volume fraction (ECV)^12^. CCT can complement echocardiography and cardiovascular magnetic resonance (CMR).

In this study, we used CCT combined with 3D technology to build an LV model to evaluate the different myocardial hypertrophy impacts on prognosis in children.

## Methods

### Study design

This is a retrospective cohort study in a larger national cardiac center. It encompasses children aged younger than 18 years of age diagnosed with HOCM at our center from April 2015 to May 2023. All underwent standard modified Morrow procedure and were evaluated for treatment outcomes by echocardiography. Clinical and basic information was obtained through a comprehensive reviewing electronic medical record (EMR), clinic records, and follow-up visits. This study was conducted in accordance with the Declaration of Helsinki and approved by the Institutional Review Board of the Ethics Committee at our Hospital (IRB NO:2022-1727). Due to the study’s retrospective design, the requirement for informed consent was waived. In addition, this study has been reported in accordance with the strengthening the reporting of cohort, cross-sectional, and case–control studies in surgery (STROCSS) criteria^13^ (Supplemental Digital Content 1, http://links.lww.com/JS9/C813).

### Patient

The inclusion criteria for this study comprised a diagnosis of HOCM, including (I) unexplained LV hypertrophy with a maximum ventricular wall thickness of >13 mm (or the equivalent of a *z*-score in pediatric patients); (II) concomitant LVOTO or corresponding symptoms^1^. Children with myocardial hypertrophy for hemodynamic or physiologic causes, a history of cardiac surgery, and other congenital heart diseases were excluded. Noonan syndrome was defined using the diagnostic criteria proposed by Dutch scholars in 1994^14^ (Supplementary Table S1, Supplemental Digital Content 2, http://links.lww.com/JS9/C814). Patients meeting these criteria were meticulously selected to ensure homogeneity in the study population.

### Cardiac CT

Sixteen of them in the cohort underwent CCT preoperatively. The CCT scan range included the whole thorax in all patients. Oral chloral hydrate (50 mg/kg) sedation is used to complete the CCT in some uncooperative children. To reduce the CT radiation dose, a dose regimen adapted to the size of the human body was used^15^. To maximize the radiation dose efficiency and the contrast-to-noise ratio of iodine, 70 kV tube voltage was chosen to prevent substantial tube current saturation, which would lead to a reduction in the image quality. Moreover, it was ensured that all radiation exposure was within safe limits for children.

### Echocardiography

Echocardiography includes transthoracic and intraoperative transesophageal measurements. The maximal LVOT gradient was measured at rest with continuous Doppler and during a Valsalva maneuver when possible. LVOTO was defined as a maximal gradient of 30 mmHg or greater. All postoperative echocardiography was completed on the day the patient was discharged from the hospital. In this study, observers of echocardiography were blinded to clinical and genetic data.

### Operation

All children with HOCM in this study underwent a septal myectomy procedure, performed by the same surgeon. The surgery involved a standard median sternotomy approach. Intraoperative transesophageal echocardiography was routinely used. The surgical technique involved making an oblique aortotomy towards the middle of the noncoronary sinus. Septal dissection was initiated at the bottom of the right aortic sinus using a scalpel and was continued towards the left, reaching the mitral valve. Resection extended towards the hinge of the mitral valve and apically to the bases of the papillary muscles. This approach can create a wider trough at the mid-ventricular level to relieve LVOTO.

### Genetic test

Genetic testing was conducted at all sites over time using various available platforms. All patients received genetic counseling and underwent analysis of 49 pediatric cardiomyopathies-associated gene panels (Supplementary Table S2, Supplemental Digital Content 2, http://links.lww.com/JS9/C814). Among them, the identified mutations were classified according to the American College of Medical Genetics and Genomics (ACMG) guidelines^16^, with only those deemed pathogenic or likely pathogenic being reported as positive gene tests in patients.

### Follow-up

Follow-ups are conducted by a professional team. They collected data on death, HF, ventricular arrhythmic composite, and recurrent LVOTO through a comprehensive review of EMR and telephone contact with doctors. The end of follow-up was set as 1 November 2023. No patients were lost to follow-up. The primary endpoint included death, HF, ventricular arrhythmic composite, and recurrent LVOTO. Ventricular arrhythmic composite was defined as patients experiencing sudden cardiac death, resuscitated cardiac arrest, or appropriate implantable cardioverter-defibrillator therapy. HF composite was defined as patients who appear with LV ejection fraction (EF) <35%, New York Heart Association (NYHA) class III/IV symptoms, heart transplantation (HTx), or left ventricular assist device (LVAD) implantation. Recurrent LVOTO refers to obstruction occurring (instantaneous peak Doppler left ventricular (LV) outflow tract pressure gradients >30 mmHg) at least 60 days after surgery^17,18^.

### Constructing 3D ventricular models

We employed a 3D modeling approach to depict the heart using ITK-SNAP Medical Image Segmentation Tool 4.0, executed by a team of professionals who were blinded to prognostic outcomes. The LV was partitioned into 17 zones following the standard approach^19^:1. basal anterior, 2. basal anterosepral, 3. basal inferoseptal, 4. basal inferior, 5. basal inferolateral, 6. basal anterolateral, 7. mid anterior, 8. mid anteroseptal, 9. mid inferoseptal, 10. mid inferior, 11. mid inferolateral, 12. mid anterolateral, 13. apical anterior, 14. apical septal, 15. apical inferior, 16. apical lateral, and 17. apex. Utilizing the software, we meticulously measured the thickness of each LV segment and computed the average thickness across basal, mid, and apical regions. Subsequently, we compared these specific ventricular thicknesses against corresponding segments, enabling an objective evaluation of hypertrophy severity across different regions. Employing cluster analysis, we correlated the 17 segments with poor prognosis, identifying hypertrophic segments linked to prognosis, and established cut-off values for high-risk and low-risk adverse prognosis. Ultimately, a comprehensive 3D LV model encapsulated our findings.

### Statistical analysis

Descriptive statistics were used to summarize categorical variables as numbers (percentages), mean±SD, or median (interquartile range, IQR). Fisher’s exact test was employed to compare categorical variables, while continuous variables were compared using either the student’s *t*-test or the Mann–Whitney *U* test. Univariate analyses were conducted using the Cox proportional hazards model. Statistical significance was defined as *P*<0.05, within a 95% CI. The margin of error was calculated by the critical value multiplying the SD. Power calculations were computed analysis using G-power software (version 3.1.9.2). All study populations were younger than 18 years old and Asian with Han Chinese ethnicity. All statistical analyses were performed using SPSS Statistics 24.0 software (IBM Corp). Data visualization was carried out using the GraphPad Prism program (version 8.0; GraphPad).

## Results

### Patient characteristics

A total of 57 children were included in the cohort, with 34 (59.6%) being males. The middle age at first diagnosis was 5 years (IQR: 1.2–8.8 years). Nine (15.8%) children were diagnosed with Noonan syndrome. Approximately half of the children (25, 43.9%) carried mutation genes associated with childhood cardiomyopathy, indicating its significant prevalence in the cohort^7^. The preoperative EF middle at 72% (IQR: 62–82%). Preoperative maximal gradients of LVOT at rest measured 80 mmHg (IQR: 47–113 mmHg), with a maximum LVOT flow of 4.4 m/s (IQR: 3.5–5.3 m/s). The middle septal thickness was 17 mm (IQR: 10–24 mm). SAM was observed in the majority, with 51 out of 57 children (89.5%) exhibiting varying degrees of it. Additionally, mid-ventricular obstruction was present in 21 out of 57 patients (36.8%) (Table 1).

**Table 1:** Patient characteristics and follow-up outcomes (*n*=57)

Variables and prognosis	All (*n*=57)
Male sex (%)	34 (59.6)
Ages of diagnosis (years)	5 (1.2–8.8)
Age at procedure less than 2 years (%)	12 (21.1)
Genetic mutation (%)	25 (43.9)
Noonan syndrome (%)	9 (15.8)
Preoperational ejection fractions (%)	72 (62–82)
Preoperational maximal gradients of LVOT at rest (mmHg)	80 (47–113)
LVOT peak flow velocity (m/s)	4.4 (3.5–5.3)
Maximum septal thickness (mm)	17 (10–24)
Left ventricle posterior wall thickness (mm)	9 (5–13)
Preoperational SAM (%)	51 (89.5)
Mid-ventricular obstruction (%)	21 (36.8)
Follow-up time (months)	36 (10–62)
Death (%)	3 (5.3)
HF (%)	4 (7)
Life-threatening arrhythmia (%)	7 (12.3)
Recurrent LVOTO (%)	10 (17.5)
Adverse events (%)	17 (29.8)

Values are *n* (%), or median (IQR). Left ventricular outflow tract

HF, heart failure; LVOTO, left ventricular outflow tract obstruction; SAM, systolic anterior motion.

### Postoperative outcomes

**Figure 1:**
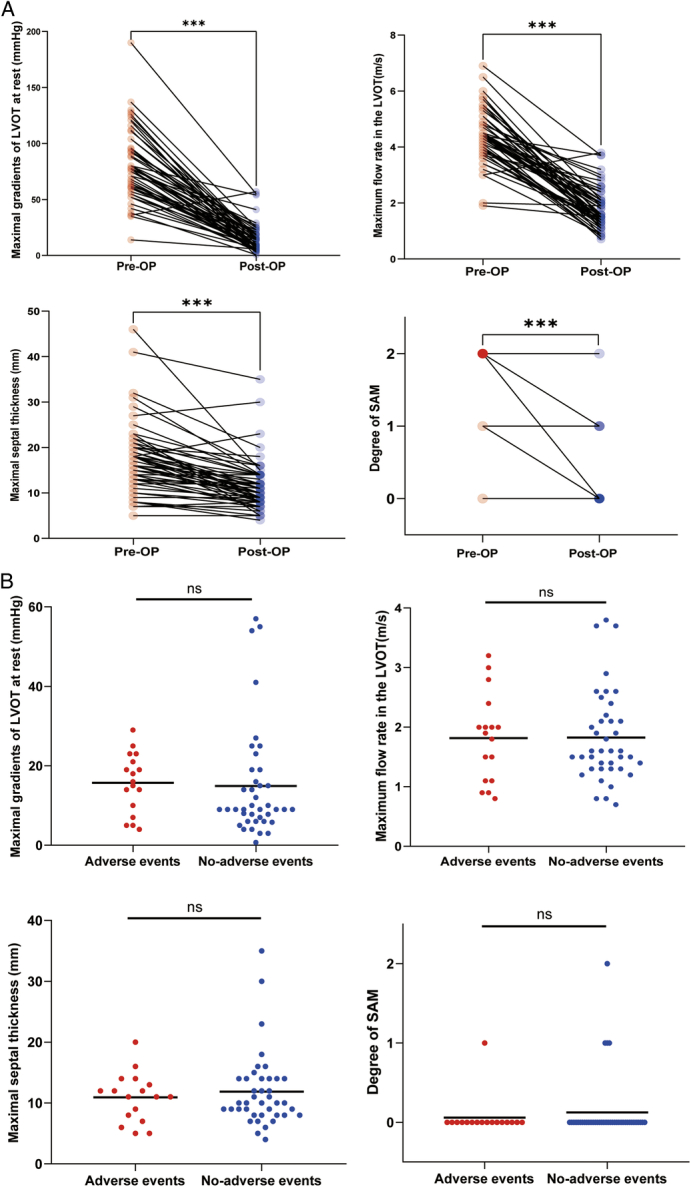
(A) comparison of changes in relevant clinical indications before and after surgery. (B) comparison of changes in relevant postoperative clinical indications between adverse events and no adverse events. Adverse event definitions include death, heart failure (HF), ventricular arrhythmic composite, and recurrent left ventricular outflow tract obstruction (LVOTO). Ventricular arrhythmic composite was defined as patients experiencing sudden cardiac death (SCD), resuscitated cardiac arrest, or appropriate implantable cardioverter-defibrillator (ICD) therapy. HF composite was defined as patients who appear with LV ejection fraction (EF) <35%, New York Heart Association (NYHA) class III/IV symptoms, heart transplantation (HTx), or left ventricular assist device (LVAD) implantation. Recurrent LVOTO refers to obstruction occurring (instantaneous peak Doppler left ventricular outflow tract pressure gradients >30 mmHg) at least 60 days after surgery. SAM, systolic anterior motion; LVOT, left ventricular outflow tract. Pre-Op: pre-operation; Post-op: post-operation. Degree of SAM:0: none, 1: partial, 2: complete.

**Figure 2:**
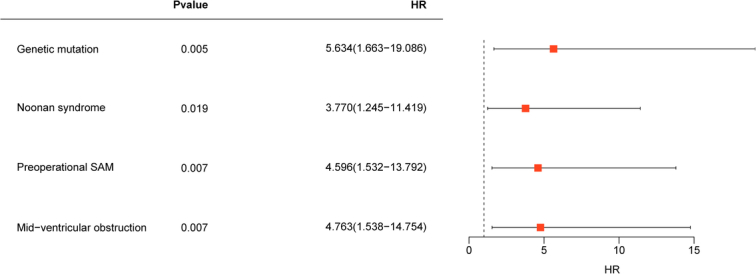
Cox multivariate retrospective analysis of forest plots. Genetic mutation, Noonan syndrome, Preoperational SAM, and Mid-ventricular obstruction are highly associated with poor prognosis in pediatric HOCM. SAM, systolic anterior motion.

**Figure 3:**
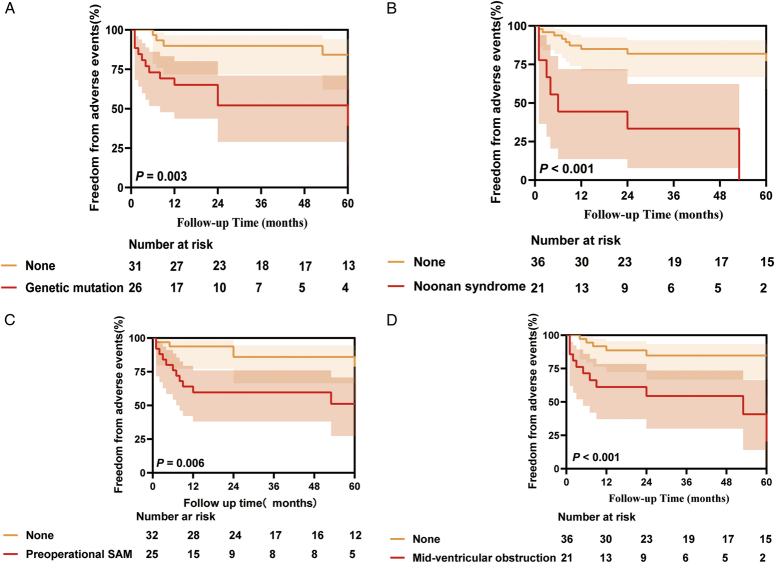
K-M curves for Genetic mutation, Noonan syndrome, Preoperational SAM, and Mid-ventricular obstruction are associated with adverse events. SAM, systolic anterior motion.

The mean follow-up time for the cohort was 36 months (IQR: 10–62 months), during which no patients died in the hospital. However, 3 (5.3%) children succumbed to HF during the follow-up period. Additionally, 17 (29.8%) children experienced endpoint events. These events included 4 (7%) cases of HF, 7 (12.3%) instances of life-threatening arrhythmic events, and recurrence of LVOTO in 10 (17.5%) cases (Table 1). These findings align with previous studies, reinforcing the significance of these outcomes^20^. Comparative analysis revealed notable improvements in relevant clinical indicators postsurgery, including LVOT pressure, flow velocity, SAM, and septal thickness (*P*<0.001, Fig. 1A). Cohort stratification based on adverse events did not reveal significant differences in relevant clinical indicators between the groups (Fig. 1B), excluding the possibility of incomplete resection as a confounding variable. Further examination of preoperative characteristics identified sex (*P*=0.015), genetic mutation (*P*=0.039), Noonan syndrome (*P*=0.002), preoperative maximal gradients of LVOT at rest (*P*=0.032), and mid-ventricular obstruction (*P*=0.007) as high-risk adverse prognostic factors (Table 2). Multivariate Cox regression analysis corroborated these findings, identifying genetic mutation (HR: 5.634, 95% CI: 1.663–19.086, *P*=0.005), preoperative SAM (HR: 4.596, 95% CI: 1.532–13.792, *P*=0.007), mid-ventricular obstruction (HR: 4.763, 95% CI: 1.538–14.754, *P*=0.007), and Noonan syndrome (HR: 3.770, 95% CI: 1.245–11.419, *P*=0.019) as high-risk factors for poor prognosis (Fig. 2, Supplementary Table S3, Supplemental Digital Content 2, http://links.lww.com/JS9/C814). Kaplan–Meier curve analysis yielded consistent results (Fig. 3). Notably, besides genetic mutations, variability in ventricular structure also correlated with poor prognosis, although the specific aspects of LV hypertrophy contributing to this outcome remain unclear.

**Table 2:** Variables between adverse events and no adverse events (*n*=57)

Variables	No-adverse events (*n*=40)	Adverse events (*n*=17)	*P*
Male sex (%)	28 (70.0)	6 (35.3)	**0.015**
Ages of diagnosis (years)	5 (0–10)	5 (0–10)	0.219
Age at procedure less than 2 years (%)	7 (17.5)	5 (29.4)	0.478
Genetic mutation (%)	14 (35.0)	11 (64.7)	**0.039**
Noonan syndrome (%)	7 (41.2)	2 (5.0)	**0.002**
Preoperational ejection fractions (%)	71 (20)	72 (20)	0.586
Preoperational maximal gradients of LVOT at rest (mmHg)	64 (42–86)	80 (35–125)	**0.032**
LVOT peak flow velocity (m/s)	4.4 (3.3–5.5)	4.2 (3.4–5.0)	0.282
Maximum septal thickness (mm)	16 (8–22)	19 (12–26)	0.108
Left ventricle posterior wall thickness (mm)	9 (4–14)	8 (4–12)	0.623
Preoperational SAM (%)	3 (50.0)	18 (35.3)	0.659
Mid-ventricular obstruction (%)	10 (25)	11 (64.7)	**0.007**

Values are *n* (%), or median (IQR).

HF, heart failure; LVOT, left ventricular outflow tract; LVOTO, left ventricular outflow tract obstruction; SAM, systolic anterior motion

### 3D model analysis outcomes

**Figure 4:**
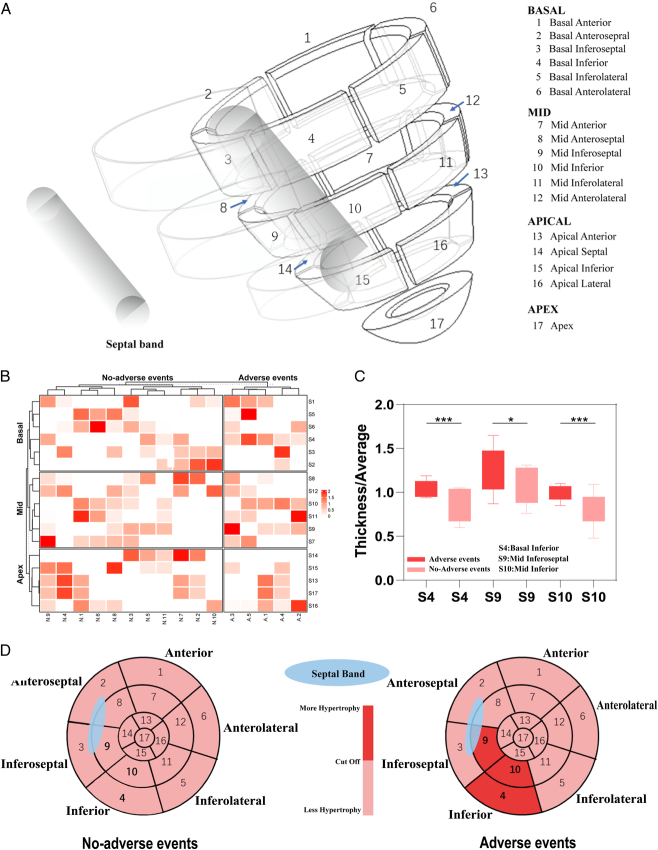
3D model of standard 17 segments in left ventricle. (A) The left ventricle was divided into: 1. Basal Anterior, 2. Basal Anterosepral, 3. Basal Inferoseptal, 4. Basal Inferior, 5. Basal Inferolateral, 6. Basal Anterolateral, 7. Mid Anterior, 8. Mid Anteroseptal, 9. Mid Inferoseptal, 10. Mid Inferior, 11. Mid Inferolateral, 12. Mid Anterolateral, 13. Apical Anterior, 14. Apical Septal, 15. Apical Inferior, 16. Apical Lateral, 17. Apex. (B) Heat map clustering analysis of the ratio of 17 segments thicknesses/average revealed an association between S4, S9, and S10 values and poor prognosis. (C) *t*-test results similarly found an association between S4, S9, and S10 values and poor prognosis. (D) We plotted the location of the S4, S9, and S10 in a transverse section of the heart by a bull’s-eye diagram, these three regions are located in the posterior wall of the left ventricle. We distinguished the high-risk poor prognosis from the low-risk poor prognosis group by cut-off values. Cut-off values: S4:0.885, S9:1.000, S10:1.330.

**Figure 5:**
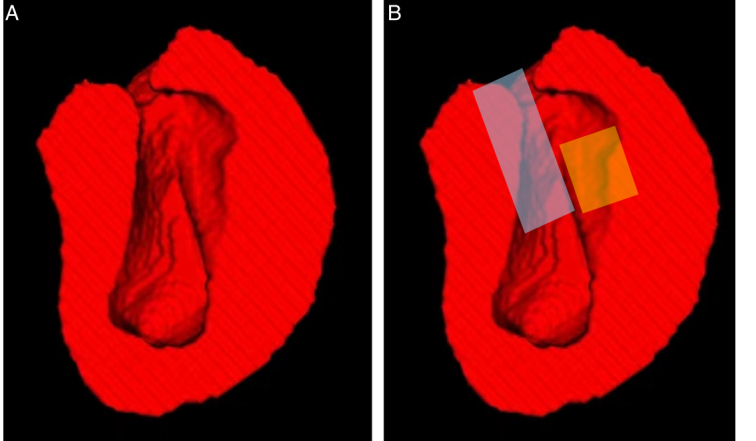
(A) Three-dimensional cardiac model of childhood hypertrophic cardiomyopathy in a high-risk adverse prognosis. (B) The septal band (blue) is a characteristic landmark that can be used to guide modified Morrow operation. The operator begins below the left coronary ring, and this band runs obliquely in a clockwise fashion down the septum to the base of the posterior medial papillary muscle. The yellow area is the posterior wall of the left ventricle, which shows the limitations of the modified Morrow procedure for resection of this area. The conduction beam is located above the yellow area. Incomplete or excessive resection here may lead to postoperative recurrent obstruction, conduction block, and other adverse events.

We present the results of the segmentation of 17 parts of the LV by 3D views (Fig. 4A). All children in our cohort underwent preoperative CCT, but 41 of them had already CCT in a local hospital before coming to our center. To minimize the influence of other confounding factors on the construction of the 3D model, these 41 children were not included. We also compared the variables between 16 children (with a 3D model) and 57 children, there was no difference between them (Supplementary Table S4, Supplemental Digital Content 2, http://links.lww.com/JS9/C814). We did this by meticulously analyzing the preoperative CCT of 16 children. These 17 segment-specific ventricular wall thicknesses were compared with the mean of the segments in which they were located (Supplementary Table S5, Supplemental Digital Content 2, http://links.lww.com/JS9/C814), and these ratios were clustered with the prognostic results, which revealed that the S4 (Basal Inferior), S9 (Mid Inferoseptal), and S10 (Mid Inferior) had a significant correlation with the prognosis (Fig. 4B). Further comparison of the results by statistical methodology was consistent with the cluster analysis (S4:*P*=0.014, S9:*P*=0.044, S10:*P*=0.017) (Fig. 4C). We set the ratio of each of the three segments cut off according to the model results (S4:0.885, S9:1.000, and S10:1.330). According to the cut-off value, we plotted a polar plot and located the 17 segments. It can be visualized that these three segments are in the posterior wall of the LV from the diagram (Fig. 4D). Therefore, we believe that there is a prognostic correlation with the different hypertrophy of the posterior wall in the LV. Further, when we reduce these 17 parts into a 3D whole LV, we can more visually see that the hypertrophy of the posterior wall, which affects the prognosis, is exactly adjacent to the region of the septal band and below the conduction bundle (Fig. 5A, B).

## Discussion

Although modified Morrow surgery is one of the preferred therapeutic options for the treatment of HOCM in children, some still have poor postoperative prognosis, including HF, recurrent LVOTO, and malignant arrhythmic events^20^. Previous studies have focused on the prognostic impact of genetic mutations and surgical efficacy, but few have addressed the prognostic impact of variability in ventricular structure^21^. In this study, we attempted to construct a 3D model of ventricular structure in children with HOCM based on CCT to study poor prognosis. To achieve this, we separated the LV into 17 segments according to standard methods^19^. The 3D model analysis revealed differences in the hypertrophy of the basal inferior, the mid inferoseptal, and the middle inferior, which may contribute to the poor prognosis (Fig. 4). For the first time, we utilized CCT to construct a 3D model to analyze the poor prognosis from a structural view. This innovative approach can guide clinicians in treating high-risk children earlier and operating aggressively.

Our study found that Noonan syndrome (HR: 3.770, 95% CI: 1.245–11.419, *P*=0.019) was a high-risk factor for poor prognosis (Fig. 2, Supplementary Table S3, Supplemental Digital Content 2, http://links.lww.com/JS9/C814). Previous reports have also demonstrated that children with Noonan syndrome carry genetic mutation and have a poor prognosis in HOCM^22^. Noonan syndromes belong to a newly categorized family of autosomal dominant syndromes called ‘RASopathies’^23^. Because the RAS/MAPK signaling pathway is prevalent in a wide range of normal cells and RAS proteins control multiple cellular processes and multiple downstream effectors, enhanced ERK1/2 signaling has been associated with concentric hypertrophy^24^. This could be a potential mechanism for poor prognosis.

Since the middle inferoseptal, and the middle inferior with hypertrophic differences are in the mid-ventricular, we can assume that the variability of mid-ventricular obstruction is an important factor affecting prognosis. Moreover, 80% of poor prognosis children carry the genetic mutation (*P*=0.052, Table 3). There are reports illustrating the presence of cardiomyopathy-associated genetic variants in nearly half of the mid-ventricular obstruction hypertrophies^25^. The variations in cardiac hypertrophy serve as a phenotype in the mutation. Several teams have reported that HCM patients with mid-ventricular obstruction (HCM-MVO) subtype have worse outcomes than patients with a common type of HCM^26^. However, this difference in LV posterior wall thickness could not be accurately identified by echocardiography (*P*=0.320, Table 3). There was no difference in age between the two groups, ruling out the effect of age on the thickness of the heart (*P*=0.743, Table 3). Through CCT combined with 3D model construction, it was found that the middle inferior of the LV was significantly associated with poor prognosis (Fig. 4B, C, D). This finding underscores the potential of CCT examination as a supplementary tool for preoperative condition assessment, enhancing the prospects of precision medicine^1^. Besides, the LV posterior wall is a part of LVOT, myocardial hypertrophy will affect the effect of obstruction relief after surgery even leading to recurrent obstruction. Therefore, for children with severe thickening of the LV posterior wall, surgeons may need to take more aggressive resection methods to avoid postoperative adverse events.

**Table 3:** Comparison of basic data for adverse event group in a 3D model prediction cohort (*n*=16)

	No-adverse events (*n*=11)	Adverse events (*n*=5)	*P*
Male sex (%)	6 (54.5)	1 (20.0)	0.308
Ages of diagnosis (years)	5 (0.5–9.5)	6 (2.5–9.5)	0.743
Age at procedure less than 2 years (%)	3 (27.3)	2 (40.0)	0.622
Genetic mutation (%)	2 (18.2)	4 (80)	**0.052**
Noonan syndrome (%)	0 (0)	1 (20.0)	0.313
Preoperational maximal gradients of LVOT at rest (mmHg)	61 (36)	77 (56)	0.510
Preoperational SAM (%)	3 (27.3)	2 (40.0)	0.516
Mid-ventricular obstruction (%)	1 (9.1)	4 (80.0)	**0.013**
Preoperational ejection fractions (%)	72 (67–77)	72 (55–89)	0.510
Preoperational maximal gradients of LVOT at rest (mmHg)	77 (49–105)	61 (43–79)	0.510
LVOT peak flow velocity (m/s)	4 (3.2–4.8)	4 (3.4–4.6)	0.743
Maximum septal thickness (mm)	16 (11–21)	20 (14–26)	**0.069**
Left ventricle posterior wall thickness (mm)	9 (6–12)	9 (3–15)	0.320

Values are *n* (%), or median (IQR).

LVOT, left ventricular outflow tract; SAM, systolic anterior motion.

Surgeons should be more cautious in the surgical treatment of children with abnormal LV posterior wall hypertrophy. The modified Morrow operation has since been further modified by an extended myectomy toward the cardiac apex, which was subsequently extended laterally as well as widened toward the mitral valve. This extended myectomy results in a more comprehensive muscle resection^27^. The entire procedure is marked by resection of the septal band, including a portion of the posterior wall of the LV. Hypertrophic myocardial resection in this region is somewhat specific, as the conduction bundle passes through this area, it is necessary to avoid the membranous portion of the interventricular septum during resection, and myocardial resection is generally performed to the right and extended to the base of the posterior medial papillary muscle while maintaining ~1.5 cm from the septum to prevent damage to the conduction system^28^. If the conduction bundle is damaged during resection it can lead to postoperative arrhythmic complications. Therefore, for children with LV posterior wall hypertrophy, surgeons should be cautious in handling the abnormal hypertrophic myocardium in this region to avoid the conduction bundle injury caused by excessive resection.

Abnormal hypertrophy of the posterior wall of the LV may also induce SAM. Previous studies have shown that the increase of LV outflow tract velocity, the length of mitral chordae tendinous, and the displacement of mitral papillary muscle can lead to SAM^29^. The thickening of the LV posterior wall will increase the LVOT of obstruction and flow velocity, which may aggravate the SAM phenomenon. Moreover, hypertrophy leads to abnormal motion of the LV posterior wall, which also plays an important role in SAM production^30^.

CCT combined with 3D model analysis can be used as a supplement to other preoperative examinations, which can better evaluate the degree of ventricular hypertrophy and provide guidance for surgical treatment. Although CMR is superior to CCT for the hypertrophic myocardium, CMR in children is mainly limited by the long examination time requiring conscious sedation or general anesthesia and the relatively low spatial resolution. Therefore, the utilization of CCT is higher than that of CMR in clinical practice. The use of advanced techniques can compensate for the shortcomings of CCT, it will be able to complement CMR. In this study, we used 3D reconstruction technology combined with CCT. It has recently been reported that CCT ventricular volumetry with semiautomatic 3D threshold-based segmentation provides highly accurate and reproducible results even in newborns and infants with congenital heart disease. It can reduce measurement errors inherent in conventional two-dimensional cardiac MRI ventricular volumetry^31^.

### Limitation

This study utilized a retrospective observational cohort design. However, the limited total cohort size included some patients with CCT, potentially impacting the robustness of the conclusions. Nevertheless, it is noteworthy that this study represents the first attempt to construct a 3D model for CCT in pediatric patients with HOCM, and the cohort benefited from comprehensive clinical and genetic data. Furthermore, being retrospective in nature, the study lacked a subsequent prospective cohort to validate the predictive accuracy of the 3D prediction model.

## Conclusion

Even after the modified Morrow surgery, the prognostic impact of a genetic mutation on childhood HOCM remains significant. In addition, we found that the degree of hypertrophy of the posterior wall in the LV was also related to the postoperative prognosis through preoperative CCT combined with 3D technology. This provides surgeons with guidance in evaluating the overall prognosis and the treatment plan before surgery.

## Ethical approval

The Institutional Review Board (IRB) of the Fuwai hospital according to the declaration of Helsinki approved the study program and the publishing of the data. Ethical Approval number: 2022-1727.

## Consent

The patients provided informed written consent for the publication of the study data.

## Source of funding

This work was supported by the National Natural Science Foundation for Distinguished Young Scholars of China [grant no. 82125004], Shenzhen Science and Technology Innovation Commission [grant no. JCYJ20220818103414030], National Natural Science Foundation of China [grant no. 82300397, 81900335, 22275124], National High Level Hospital Clinical Research Funding [grant no. 2023-GSP-QN-13, 2023-GSP-ZD-1], Guangdong Provincial Enterprise Joint Foundation [grant no. 2022A1515220103] and Program for Guangdong Introducing Innovative and Entrepreneurial Teams [grant no. 2019ZT08Y481].

## Author contribution

L.S. and H.X.M.: conceptualization, formal analysis, methodology, writing – original draft, and writing – review and editing; S.J.P. and Y.J.: conceptualization, data curation, formal analysis, methodology, and writing – review and editing; M.H., Z.Y.Q., C.X., and W.W.T.: formal 3D model and analysis, methodology, and visualization; L.Y.J. and Z.Q.: writing – review and editing.

## Conflicts of interest disclosure

The authors declare no conflicts of interest.

## Research registration unique identifying number (UIN)

Name of the registry: Clinicaltrials.gov. Unique identifying number or registration ID: NCT05687487.

## Guarantor

The guarantor of this work is Jiangping Song.

## Data availability statement

The datasets used and/or analyzed during the current study are available from the corresponding author on reasonable request.

## Provenance and peer review

The paper was not invited.

## Supplementary Material
