# Supplementary material for: Left ventricular posterior wall hypertrophy leads to poor prognosis of hypertrophic obstructive cardiomyopathy in children: a cohort study
Source: Int J Surg. 2024 Jun 21;111(1):771–80. doi: 10.1097/JS9.0000000000001862 (PMC11745586; doi:10.1097/JS9.0000000000001862)
Supplement: Supplementary file 2 [file js9-111-0771-s002.docx]

**Supplemental Digital Content**

**Table S1: Diagnostic Criteria for Noonan syndrome^14^.**

| **Feature** | **A = major** | **B = minor** |
| --- | --- | --- |
| 1. Facial | Typical face | Suggestive face |
| 2. Cardiac | Pulmonary valve stenosis and/or typical ECG | Other defect |
| 3.Height | $<$3rd centile | $<$10th centile |
| 4. Chest wall | Pectus carinatus/excavatum | Broad thorax |
| 5. Family history | First degree relative definite Noonan syndrome | First degree relative suggests Noonan syndrome |
| 6. Other | All 3(males): mental retardation, cryptorchidism, lymphatic dysplasia | One of mental retardation, cryptorchidism, lymphatic dysplasia |

Definite Noonan syndrome: 1A plus one of 2A-6A or two of 2B-6B; 1B plus two of 2A-6A or three of 2B-6B. Noonan syndrome was defined using the diagnostic criteria proposed by Dutch scholars in 1994^14^.

**Table S2 Gene testing panel associated with pediatric cardiomyopathies.**

| **Classification** | **Gene symbol** |
| --- | --- |
| Sarcomere: thin filament | *ACTC1, TNNC1, TNNI3, TNNT2, TPM1* |
| Sarcomere: thick filament | *MYBPC3, MYH7, MYL2, MYL3* |
| Z disc | *ACTN2, CSRP3, LDB3/ZASP, MYOZ2, TCAP, TTN* |
| Desmosome | *DSC2, DSG2, DSP, JUP, PKP2, TMEM43, RYR2* |
| Cytoskeletal | *VCL* |
| Intermediate filament | *DES* |
| Nuclear membrane | *EMD, LMNA, SYNE1, SYNE2* |
| Plasma membrane | *CAV3, SGCD* |
| Other | *CRYAB, MIB1, RMB20,* |
| Syndromic cardiomyopathies | *BRAF, HRAS, KRAS, PTPN11, SOS1, SPRED1* |
| Metabolic disorders | *CPT2, GAA, HADHA, LAMP2, MT-TL1, PRKAG2, SLC22A5, TAZ* |
| NMDs/neurodegenerative disorders | *DMD, FXN* |

**Table S3: Cox regression analysis of risk factors associated with poor prognosis (n=57).**

|  | **Univariate analysis** |  | **Multivariate analysis** |  |
| --- | --- | --- | --- | --- |
| **Outcomes** | **HR (95% CI)** | ***P*** | **HR (95% CI)** | ***P*** |
| Male gender | 0.320(0.118-0.870) | **0.025** | - | - |
| Genetic mutation | 3.785(1.297-11.046) | **0.015** | 5.634(1.663-19.086) | **0.005** |
| Noonan syndrome | 7.070(2.530-19.754) | **0.001** | 3.770(1.245-11.419) | **0.019** |
| Preoperational SAM | 2.951(1.169-7.454) | **0.022** | 4.596(1.532-13.792) | **0.007** |
| Mid-ventricular obstruction | 5.546(1.896-16.221) | **0.002** | 4.763(1.538-14.754) | **0.007** |

LVOT left ventricular outflow tract; SAM, systolic anterior motion.

**Table S4: variables between 16 children (with 3D model) and 57 children.**

| **Variables** | **16 children (with 3D model)** | **57 children** | ***P* value** |
| --- | --- | --- | --- |
| Male gender (%) | 7(43.8) | 34(59.6) | 0.257 |
| Ages of diagnosis (years) | 5(1-9) | 5(3-7) | 0.856 |
| Age at procedure less than two years (%) | 5(31.2) | 12(21.1) | 0.394 |
| Genetic mutation (%) | 6(37.5) | 25(43.9) | 0.649 |
| Noonan syndrome (%) | 1(6.3) | 9(15.8) | 0.569 |
| Preoperational ejection fractions (%) | 72(69.5-74.5) | 72(67-77) | 0.768 |
| Preoperational maximal gradients of LVOT at rest (mmHg) | 63(50-76) | 80(64-96) | 0.109 |
| LVOT peak flow velocity(m/s) | 4(2-6) | 4.4(3.9-4.9) | 0.140 |
| Maximum septal thickness (mm) | 17(14-20) | 17(13.5-20.5) | 0.779 |
| Left ventricle posterior wall thickness (mm) | 9(7.5-10.5) | 9(7-11) | 0.727 |
| Preoperational SAM (%) | 16(100) | 51(89.5) | 0.328 |
| Mid-ventricular obstruction (%) | 5(31.3) | 21(36.8) | 0.680 |

Values are n (%), or median (IQR). LVOT left ventricular outflow tract; SAM, systolic anterior motion; HF, heart failure; LVOTO, left ventricular outflow tract obstruction.

**Table S5:** **Mean thickness of basal, mid, and apex in the left ventricle and its 17 segments-to-mean thickness ratios(n=16).**

| **ID** | **basal average/cm** | **mid average/cm** | **apex average/cm** | **1basal anterior/average** | **2basal anterosepral/average** | **3basal inferoseptal/average** | **4basal inferior/average** | **5basal inferolateral/avergage** | **6basal anterolateral/average** | **7mid-anterior/average** | **8mid-anteroseptal/average** |
| --- | --- | --- | --- | --- | --- | --- | --- | --- | --- | --- | --- |
| 1 | 2.46 | 2.14 | 0.65 | 1.08 | 1.47 | 0.82 | 1.07 | 0.92 | 0.72 | 0.86 | 1.15 |
| 2 | 1.45 | 1.07 | 0.31 | 0.70 | 1.08 | 1.14 | 0.94 | 0.81 | 1.02 | 0.74 | 0.89 |
| 3 | 2.64 | 1.79 | 2.07 | 1.24 | 1.00 | 1.14 | 0.98 | 0.94 | 0.94 | 1.01 | 1.28 |
| 4 | 1.66 | 1.61 | 1.40 | 0.65 | 1.21 | 1.54 | 0.96 | 0.61 | 0.69 | 0.52 | 0.96 |
| 5 | 1.83 | 1.63 | 0.94 | 1.15 | 0.81 | 1.09 | 1.19 | 1.32 | 0.59 | 1.15 | 1.11 |
| 6 | 2.82 | 2.73 | 0.97 | 0.84 | 0.87 | 1.12 | 0.78 | 1.21 | 1.02 | 1.00 | 0.90 |
| 7 | 2.75 | 2.26 | 0.33 | 1.02 | 1.49 | 1.32 | 0.64 | 0.75 | 0.80 | 0.75 | 1.37 |
| 8 | 1.14 | 1.02 | 0.59 | 1.31 | 1.04 | 1.19 | 0.78 | 0.96 | 1.03 | 1.06 | 1.16 |
| 9 | 1.81 | 1.57 | 2.14 | 0.95 | 1.02 | 1.43 | 0.80 | 0.88 | 0.87 | 0.86 | 1.14 |
| 10 | 2.19 | 2.21 | 0.89 | 0.88 | 0.89 | 1.26 | 1.05 | 0.88 | 0.91 | 0.84 | 1.30 |
| 11 | 1.86 | 1.81 | 0.58 | 0.77 | 0.98 | 0.95 | 0.60 | 1.11 | 1.36 | 1.02 | 1.12 |
| 12 | 1.80 | 1.78 | 0.88 | 0.43 | 1.29 | 1.31 | 1.04 | 0.78 | 0.58 | 0.63 | 1.41 |
| 13 | 1.62 | 2.01 | 1.67 | 0.78 | 0.92 | 1.33 | 0.67 | 1.18 | 0.89 | 1.05 | 0.89 |
| 14 | 1.89 | 2.14 | 0.39 | 1.17 | 1.00 | 1.20 | 1.04 | 0.91 | 0.84 | 1.43 | 1.18 |
| 15 | 1.62 | 1.50 | 1.21 | 0.96 | 1.55 | 1.34 | 0.83 | 0.75 | 0.53 | 0.68 | 1.05 |
| 16 | 1.91 | 1.79 | 2.88 | 0.76 | 1.23 | 1.20 | 0.93 | 0.78 | 0.86 | 0.64 | 1.09 |

**Table S5: Mean thickness of basal, mid, and apex in the left ventricle and its 17 segments-to-mean thickness ratio(n=16).**

| **ID** | **9mid-inferoseptal/average** | **10mid-inferior/average** | **11mid-inferolateral/average** | **12mid-anterolateral/average** | **13apical anterior/average** | **14apical septal/average** | **15apical inferior/average** | **16apical lateral/average** | **17apex average/cm** |
| --- | --- | --- | --- | --- | --- | --- | --- | --- | --- |
| 1 | 1.21 | 1.03 | 0.76 | 0.85 | 1.27 | 0.97 | 0.84 | 1.19 | 1.07 |
| 2 | 1.30 | 0.99 | 1.32 | 0.85 | 0.64 | 0.80 | 0.82 | 1.38 | 0.91 |
| 3 | 1.65 | 0.85 | 0.95 | 1.05 | 0.91 | 0.97 | 1.00 | 1.03 | 0.98 |
| 4 | 1.20 | 1.10 | 0.81 | 0.92 | 1.08 | 0.90 | 1.04 | 1.06 | 1.02 |
| 5 | 0.87 | 1.04 | 0.98 | 1.00 | 0.93 | 1.06 | 0.91 | 1.04 | 0.98 |
| 6 | 1.04 | 1.09 | 1.31 | 0.67 | 1.16 | 0.99 | 0.76 | 1.25 | 1.04 |
| 7 | 0.76 | 0.67 | 0.72 | 0.85 | 1.08 | 1.42 | 0.89 | 0.68 | 1.02 |
| 8 | 1.31 | 0.92 | 0.60 | 1.01 | 0.67 | 1.52 | 0.57 | 0.91 | 0.92 |
| 9 | 0.79 | 0.48 | 0.73 | 1.12 | 1.35 | 0.81 | 1.21 | 0.98 | 1.09 |
| 10 | 1.30 | 0.72 | 0.68 | 0.94 | 0.64 | 1.32 | 0.97 | 0.71 | 0.91 |
| 11 | 0.90 | 0.98 | 1.09 | 0.91 | 0.92 | 1.00 | 0.92 | 1.08 | 0.98 |
| 12 | 0.88 | 0.70 | 0.53 | 1.09 | 1.18 | 1.63 | 0.59 | 0.79 | 1.04 |
| 13 | 1.19 | 0.95 | 0.90 | 1.07 | 0.79 | 0.59 | 1.30 | 1.11 | 0.95 |
| 14 | 1.15 | 0.74 | 0.86 | 1.06 | 1.11 | 0.74 | 1.16 | 1.10 | 1.03 |
| 15 | 0.98 | 0.59 | 0.57 | 1.17 | 0.94 | 1.03 | 0.77 | 1.20 | 0.99 |
| 16 | 1.28 | 0.93 | 0.91 | 0.80 | 0.82 | 1.19 | 0.98 | 0.84 | 0.95 |
